# Melatonin Mitigates Atrazine-Induced Renal Tubular Epithelial Cell Senescence by Promoting Parkin-Mediated Mitophagy

**DOI:** 10.34133/research.0378

**Published:** 2024-05-17

**Authors:** Yu-Sheng Shi, Tian-Ning Yang, Yu-Xiang Wang, Xiang-Yu Ma, Shuo Liu, Yi Zhao, Jin-Long Li

**Affiliations:** ^1^ College of Veterinary Medicine, Northeast Agricultural University, Harbin 150030, P.R. China.; ^2^ Key Laboratory of the Provincial Education Department of Heilongjiang for Common Animal Disease Prevention and Treatment, Northeast Agricultural University, Harbin 150030, P.R. China.; ^3^ Heilongjiang Key Laboratory for Laboratory Animals and Comparative Medicine, Northeast Agricultural University, Harbin 150030, P.R. China.

## Abstract

The accumulation of senescent cells in kidneys is considered to contribute to age-related diseases and organismal aging. Mitochondria are considered a regulator of cell senescence process. Atrazine as a triazine herbicide poses a threat to renal health by disrupting mitochondrial homeostasis. Melatonin plays a critical role in maintaining mitochondrial homeostasis. The present study aims to explore the mechanism by which melatonin alleviates atrazine-induced renal injury and whether parkin-mediated mitophagy contributes to mitigating cell senescence. The study found that the level of parkin was decreased after atrazine exposure and negatively correlated with senescent markers. Melatonin treatment increased serum melatonin levels and mitigates atrazine-induced renal tubular epithelial cell senescence. Mechanistically, melatonin maintains the integrity of mitochondrial crista structure by increasing the levels of mitochondrial contact site and cristae organizing system, mitochondrial transcription factor A (TFAM), adenosine triphosphatase family AAA domain-containing protein 3A (ATAD3A), and sorting and assembly machinery 50 (Sam50) to prevent mitochondrial DNA release and subsequent activation of cyclic guanosine 5′-monophosphate–adenosine 5′-monophosphate synthase pathway. Furthermore, melatonin activates Sirtuin 3–superoxide dismutase 2 axis to eliminate the accumulation of reactive oxygen species in the kidney. More importantly, the antisenescence role of melatonin is largely determined by the activation of parkin-dependent mitophagy. These results offer novel insights into measures against cell senescence. Parkin-mediated mitophagy is a promising drug target for alleviating renal tubular epithelial cell senescence.

## Introduction

Human longevity is increasing with the improvement of medical technology and living conditions. It is predicted that the number of elderly people may exceed 1.4 billion by 2050 [1]. Population aging is becoming a global challenge, as the increased lifespan of humans leads to more late-life diseases [2]. The senescent cells in various organs are considered to contribute to age-related diseases and organismal aging, including nutrient absorption defects in gut and liver fibrosis [3,4]. Many studies have shown that clearing senescent cells from the body is beneficial for maintaining organism health. Cell senescence is defined as irreversible proliferative arrest characterized by enhanced activity of senescence-associated β-galactosidase (SA-β-gal) and senescence-associated secretory phenotype (SASP) release [5]. Recent research has demonstrated that cellular senescence can be induced by oxidative stress and mitochondrial dysfunction [6]. However, effective ways to alleviate cellular senescence need to be further explored.

The maintenance of kidney functions requires a lot of energy supplied by mitochondria. The kidney contains the second highest number of mitochondria, which are located in the renal tubules [7]. Mitophagy is an important process in maintaining mitochondrial homeostasis [8]. Activated PTEN-induced kinase 1 (pink1)–parkin-mediated mitophagy has been proven to decrease the production of reactive oxygen species (ROS), thereby alleviating renal injury [9]. Recent evidence suggested that mitophagy also plays a main role in cellular senescence process. Activated parkin attenuated ROS levels and cellular senescence in airway epithelial cells [10]. The integrality of mitochondria cristae is also involved in maintaining mitochondrial homeostasis. Recent studies demonstrated that the structure destroying of mitochondria cristae induced inflammation and kidney injury through leaked mitochondrial DNA (mtDNA) [11]. Cyclic guanosine 5′-monophosphate–adenosine 5′-monophosphate synthase (cGAS) can be activated by leaked mtDNA in the cytoplasm. It has been proven that cGAS activates stimulator of interferon genes (STING) and promotes inflammatory response, which has been considered a basic drive of cellular senescence [12]. It has recently been reported that the activation of a noncanonical cGAS–STING–protein kinase R (PKR)-like endoplasmic reticulum kinase (PERK)–eukaryotic initiation factor-2α (eIF2α) axis can also drive cellular senescence [13]. However, it remains unclear whether melatonin restricts mtDNA leakage and the activation of cGAS by maintaining the integrity of mitochondrial cristae.

The application of herbicide effectively reduces the negative impact of weeds on crop yields. Most of herbicides can stably exist in the environment for an extended period and accumulate in the organism. Atrazine as a triazine herbicide is extensively used worldwide to inhibit weeds growth. The annual consumption of atrazine is greater than 9,000 tons [14]. Atrazine is found to persist in groundwater, even following its prohibition [15]. The long-term persistence of atrazine in the environment may be attributed to its better water solubility [16]. Atrazine has been banned in European Union due to its persistence, but it is still available in other areas. The application of atrazine presents a potential risk to public health. Ingestion is one of the main pathways for exposure to atrazine [17]. Recent studies have proven that atrazine exposure is associated with nephrotoxicity, reproduction toxicity immunotoxicity, and neurotoxicity [18]. However, further research is needed to elucidate the mechanism and preventive measure of atrazine-induced renal injury.

Melatonin is mainly secreted by the pineal gland and has many biological activities such as antioxidation [19]. Melatonin can protect against neuronal death by activating mitophagy and autophagy [20]. Several studies have found that melatonin ameliorates oxidative stress and promotes mitophagy through the activation of Sirtuin 3 (Sirt3) [21]. Sirt3 as a protein deacetylase located in mitochondria decreased ROS levels by activating superoxide dismutase 2 (SOD2) [22]. Targeting Sirt3 is thought to be an effective method to delay the development of cellular senescence by improving mitophagy [23]. A recent report has highlighted the protective effects of melatonin in renal injury [24]. Melatonin levels in plasma were decreased during renal injury, and higher melatonin levels are associated with reduced renal tubule damage [25]. Continuous melatonin treatment also improved mitochondrial function of aged mice [26]. It has been proven that melatonin alleviated cell senescence by improving mitochondrial function [27]. However, the mechanisms responsible for the beneficial role of melatonin and its effects on cell senescence in the kidney are still not completely known.

The kidney is an essential organ in the body that removes toxins and metabolic waste from the blood. There is growing evidence that the accumulation of senescent cells is related to kidney aging and disease, which might be a huge challenge to medical system [28]. Notably, renal tubular epithelial cell may be more susceptible to cellular senescence than other cells in the kidney. Therefore, the present study aims to focus on the protective effects of melatonin and the roles of parkin-mediated mitophagy in atrazine-induced renal tubular epithelial cell senescence. The study reveals that the antisenescence effect of melatonin relies on parkin-mediated mitophagy. Melatonin maintains the integrity of mitochondria to prevent overactivation of the cGAS–STING pathway caused by leaked mtDNA. Furthermore, activated mitophagy removes damaged mitochondria and inhibits oxidative stress. The study demonstrates that targeting parkin-mediated mitophagy is an effective approach for alleviating cellular senescence.

## Results

### Melatonin alleviates atrazine-induced renal tubular epithelial cell senescence in kidneys from wild-type mice

**Fig. 1.**
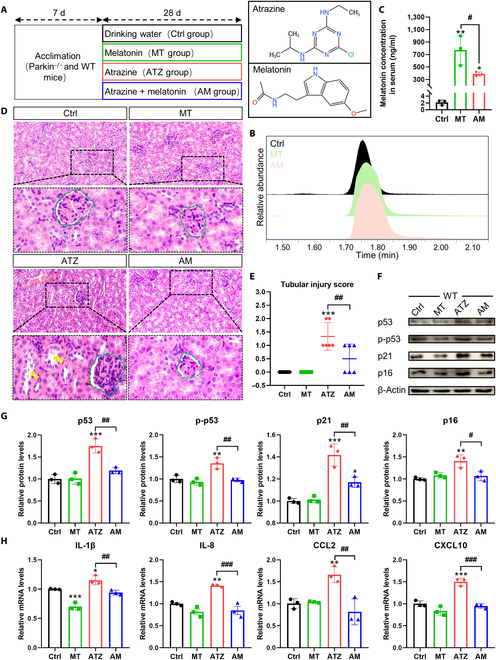
Melatonin alleviates atrazine-induced renal tubular epithelial cell senescence and injury in WT mice (*n* = 3). (A) The experimental procedures of the present research and chemical structures of atrazine and melatonin. (B) Illustrative images of serum samples analyzed using LC-MS. (C) Melatonin levels in serum. (D) H&E staining images of kidneys from different groups. Yellow arrows represent injured kidney tubulars, and green dashed line represents the glomerulus. Scale bars, 50 μm. (E) Quantitative results of kidney tubular injury. (F and G) Western blotting images and quantitative results of proteins. (H) Relative mRNA levels of SASP. Statistics data are presented as the means ± SD. Symbol for the significance of differences between the Ctrl group and another group: *P < 0.05, **P < 0.01, and ***P < 0.001. Symbol for the significance of differences between 2 groups: *#P* < 0.05, *##P* < 0.01, and *###P* < 0.001.

Liquid chromatography-mass spectrometry (LC-MS) was used to verify whether the oral gavage treatment of melatonin at a dose of 5 mg/kg per day in the study increases the concentration of melatonin in serum. Melatonin concentration was significantly increased in melatonin group (MT group) and atrazine + melatonin group (AM group) (Fig. 1B and C), which indicated that the current protocol effectively increases the melatonin levels in mouse serum. To assess the extent of renal damage following exposure to atrazine, we performed hematoxylin and eosin (H&E) staining. H&E results revealed that atrazine exposure resulted in an increased renal injury, characterized by the renal tubular dilatation, loss of brush border, and proliferation of intraglomerular mesangial cell. As expected, melatonin treatment relieved atrazine-induced renal injury. Furthermore, a tubular injury score confirmed the protective role of melatonin in against atrazine-induced injury (Fig. 1E). Enhanced expressions of p53, p21, p16, and SASP are the markers of cellular senescence. In Fig. 1F and G, compared with control (Ctrl) group, the protein levels of p53, phospho-p53 (p-p53), p21, and p16 were increased in atrazine group. Atrazine exposure also increased the mRNA expressions of SASP (Fig. 1H). Interestingly, melatonin supplementation significantly reduced the levels of p53, p-p53, p21, p16, interleukin-1β (IL-1β), IL-8, C-C motif chemokine ligand 2 (CCL2), and C-X-C motif chemokine ligand 10 (CXCL10) following exposure to atrazine (Fig. 1F to H).

**Fig. 2.**
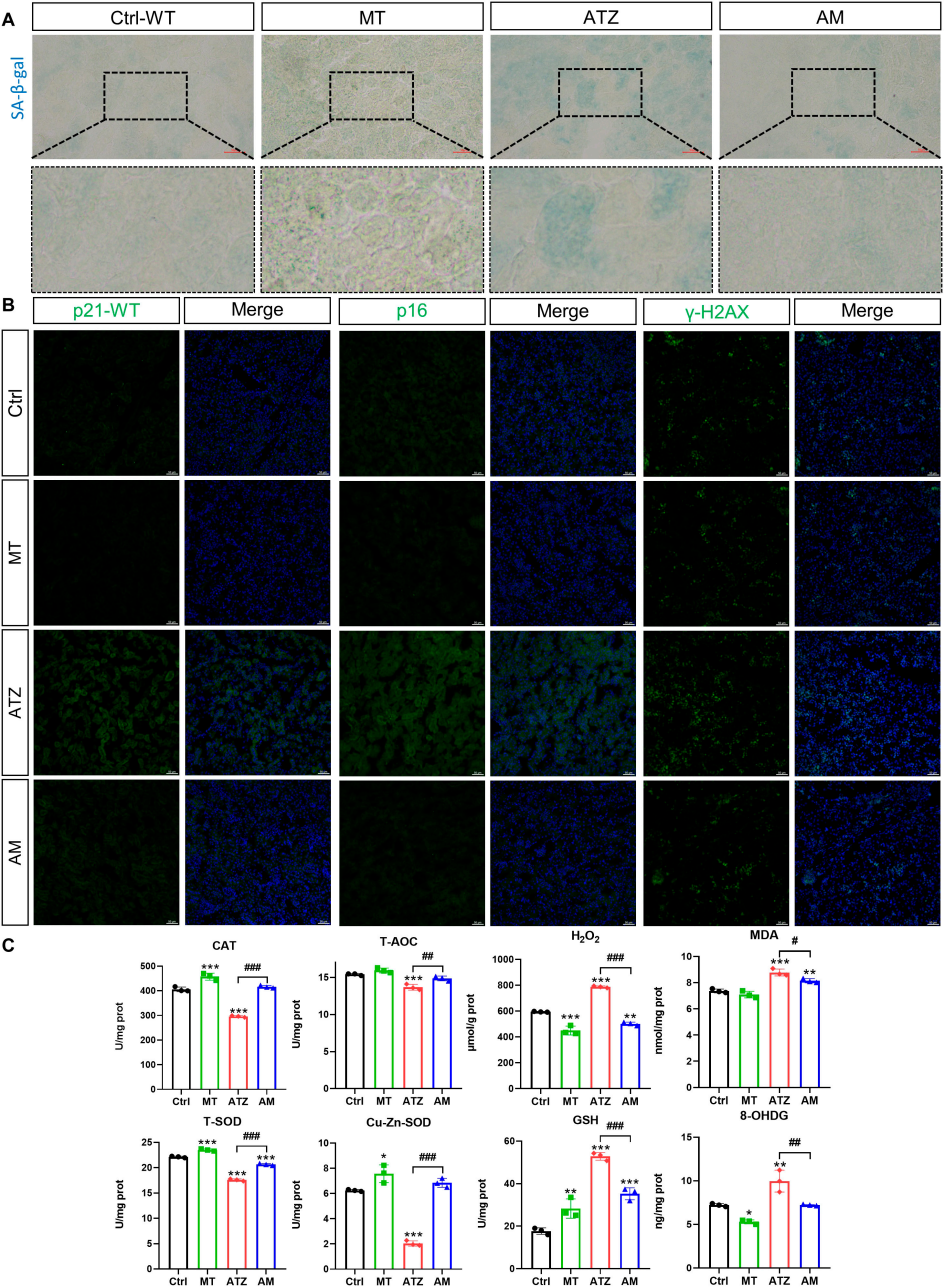
Melatonin alleviates atrazine-induced renal tubular epithelial cell senescence, DNA damage, and oxidative stress in WT mice (*n* = 3). (A) SA-β-gal staining images of kidneys from different groups. Scale bars, 50 μm. (B) Immunofluorescence images of p21, p16, γ-H2AX (green), and 4′,6-diamidino-2-phenylindole (DAPI) (blue). Scale bars, 50 μm. (C) The CAT, T-AOC, H_2_O_2_, MDA, T-SOD, Cu-Zn-SOD, GSH, and 8-OHDG levels of kidneys from different groups. Statistics data are presented as the means ± SD. Symbol for the significance of differences between the Ctrl group and another group: *P < 0.05, **P < 0.01, and ***P < 0.001. Symbol for the significance of differences between the atrazine (ATZ) group and atrazine + melatonin (AM) group: *#P* < 0.05, *##P* < 0.01, and *###P* < 0.001.

Compared with Ctrl group, SA-β-gal staining results also showed increased positively stained areas that were mainly distributed in renal tubular upon exposure to atrazine, but they were reversed by melatonin supplementation (Fig. 2A). DNA damages are relevant to cellular senescence establishment, and the expression levels of gamma H2AX (γ-H2AX) were enhanced during this period. The data also showed that melatonin supplementation reduced the number of γ-H2AX-positive cells following exposure to atrazine (Fig. 2B). Immunofluorescence staining assay of p21 and p16 showed that the positively stained areas were raised in atrazine exposure group but decreased by melatonin treatment (Fig. 2B). These data indicated that renal tubular cells are more sensitive to atrazine-induced cellular senescence, and melatonin supplementation is an effective strategy for reversing renal injury and cellular senescence.

### Melatonin inhibits oxidative stress and mitochondrial injury in kidneys from wild-type mice

**Fig. 3.**
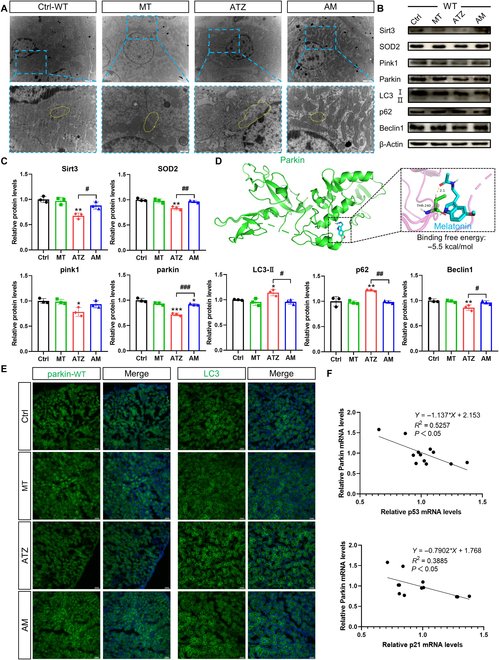
Melatonin alleviates atrazine-caused cell senescence in WT mice by improving mitophagy and Sirt3–SOD2 antioxidation axis (*n* = 3). (A) Transmission electron microscopy images of kidneys from different groups. Yellow dashed lines represent the mitochondria. (B and C) Western blotting images and quantitative results of proteins. (D) Molecular docking simulation for the binding of melatonin with parkin. (E) Immunofluorescence images of parkin, LC3 (green) and DAPI (blue). Scale bars, 50 μm. (F) Correlation between p53, p21, and parkin in kidneys of WT mice. Statistics data are presented as the means ± SD. Symbol for the significance of differences between the Ctrl group and another group: *P < 0.05, **P < 0.01, and ***P < 0.001. Symbol for the significance of differences between the ATZ group and AM group: *#P* < 0.05, *##P* < 0.01, and *###P* < 0.001.

Impaired mitochondria produce ROS, which drive cellular senescence [29]. The structures of mitochondria in the atrazine group exhibited swelling, vacuolation, and fragmentation of mitochondria and the damages of mitochondrial cristae (Fig. 3A). Sirt3 is important in protecting cells from ROS by increasing the activation of antioxidant enzymes. Compared with Ctrl group, an apparent down-regulation of the Sirt3 and SOD2 expression was shown in atrazine exposure group. The decrease in Sirt3 and SOD2 in the atrazine group was accompanied by the decreased activities of antioxidant enzymes, including catalase (CAT), total antioxidant capacity (T-AOC), and SOD (Fig. 2C). The activity of glutathione (GSH) was not affected by atrazine, and the increase in GSH in the atrazine group was reversed after melatonin treatment. Consistent with the decrease in antioxidant enzymes activity, the contents of H_2_O_2_, malondialdehyde (MDA), and 8-hydroxydeoxyguanosine (8-OHDG) were increased in the atrazine group (Fig. 2C). Notably, melatonin supplementation effectively reduced mitochondria injury and enhanced the activities of antioxidant enzymes. These data indicate that enhancing Sirt3-mediated antioxidant abilities is essential for antisenescence roles of melatonin.

### Melatonin promotes parkin-mediated mitophagy in kidneys from wild-type mice

Mitophagy eliminates impaired mitochondria from cells. Suppressed mitophagy is involved in the progression of cell senescence. The expression of LC3 was increased during atrazine exposure (Fig. 3B, C, and E). However, atrazine-induced increase in LC3 was reserved by melatonin treatment. In accordance with the accumulation of impaired mitochondria observed by transmission electron microscope, the level of sequestosome 1 (p62) was increased after atrazine exposure (Fig. 3B and C). The decrease in Beclin 1 indicated the autophagy process inhibition, but these changes were revised by melatonin (Fig. 3B and C). Parkin-dependent mitophagy is a major pathway in the maintenance of mitochondrial homeostasis. The effect of melatonin on the parkin-mediated mitophagy after atrazine exposure was investigated. In Fig. 3B, C, and E, the decrease in both pink1 and parkin induced by atrazine exposure was all attenuated by melatonin. The binding mechanism between parkin and melatonin was explored by molecular docking. As shown in Fig. 3D, the docking result revealed that parkin and melatonin had the lowest docking energy (−5.5 kcal/mol), which indicates that melatonin could combine with parkin better. The results showed the activation of mitophagy with the increased levels of pink1, parkin, and Beclin 1 and decreased expression of p62 after melatonin treatment. Overall, these results demonstrate that melatonin alleviates renal tubular cell senescence by repairing defective parkin-dependent mitophagy.

### Parkin deficiency disrupts mitophagy and aggravates renal injury

**Fig. 4.**
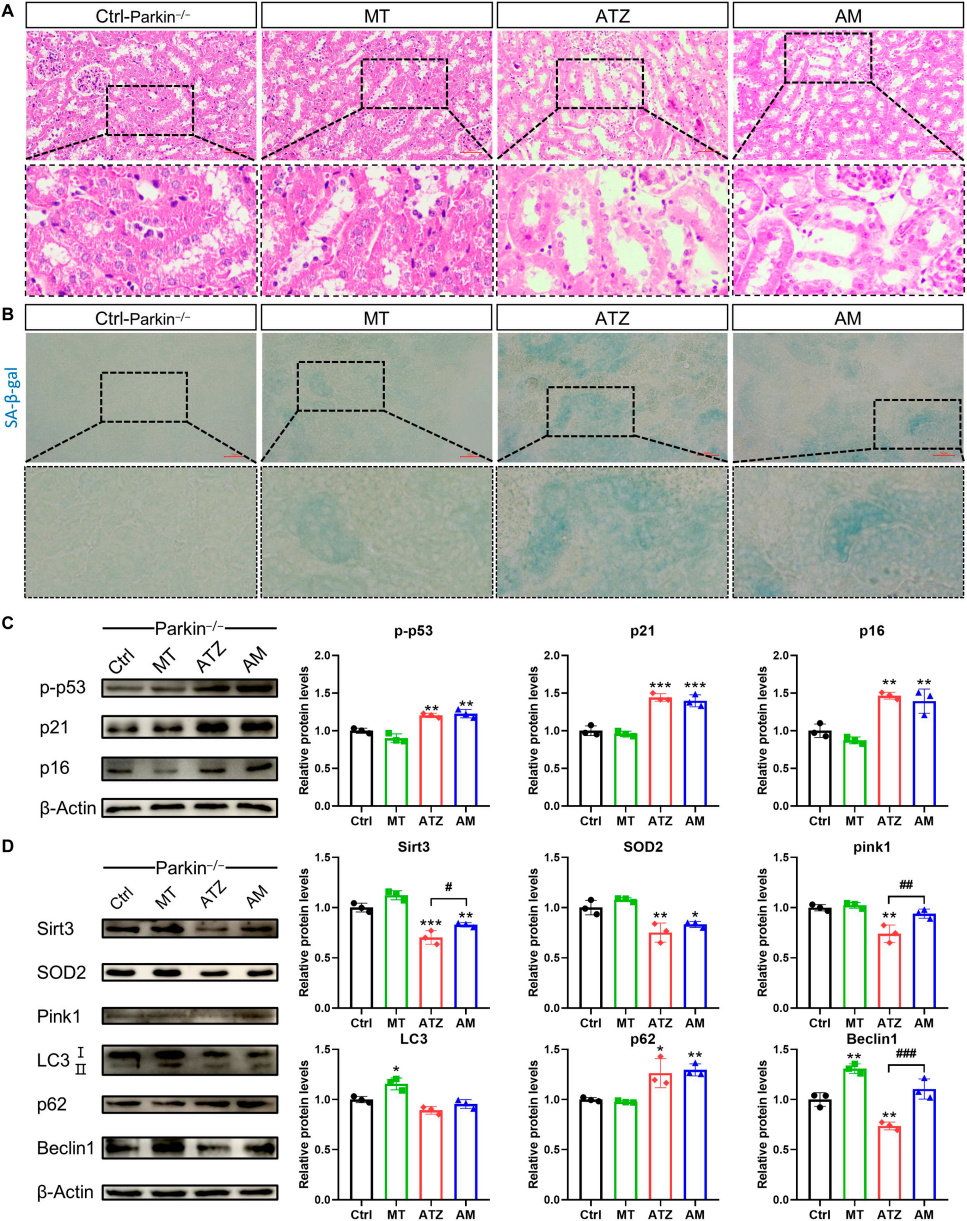
Parkin deficiency aggravates atrazine-induced kidney injury and cell senescence in parkin^−/−^ mice (*n* = 3). (A) H&E staining images of kidneys from different groups. Scale bars, 50 μm. (B) SA-β-gal staining images of kidneys from different groups. Scale bars, 50 μm. (C and D) Western blotting images and quantitative results of proteins. Statistics data are presented as the means ± SD. Symbol for the significance of differences between the Ctrl group and another group: *P < 0.05, **P < 0.01, and ***P < 0.001. Symbol for the significance of differences between the ATZ group and AM group: *#P* < 0.05, *##P* < 0.01, and *###P* < 0.001.

In Fig. 3F, a correlation analysis indicated that the parkin level was negatively correlated with the levels of p53 and p21. Given the data that the inhibition of parkin is linked to the progression of senescence, the parkin knockout (parkin^−/−^) mice were used for further identifying the role of parkin-mediated mitophagy in renal tubular epithelial cell senescence (Fig. S2). Histologic analysis revealed that parkin deficiency exacerbates atrazine-induced renal injury with severe tubular dilation in parkin^−/−^ mice and was not alleviated by melatonin supplementation (Fig. 4A). Western blotting results showed that Sirt3 and SOD2 levels were increased after melatonin treatment during atrazine exposure compared with atrazine group in parkin^−/−^ mice (Fig. 4D). There was still a decrease in Sirt3 and SOD2 levels upon melatonin treatment during atrazine exposure. The above results indicate that the Sirt3–SOD2 axis is partially affected by parkin deficiency. Then, the effects of melatonin on mitophagy in parkin^−/−^ mice during atrazine exposure were evaluated. As expected, the pink1 and LC3-II levels in atrazine and AM groups have no significant changes (Fig. 4D). The decrease in Beclin1 level by atrazine was reversed by melatonin (Fig. 4D). Furthermore, p62 levels were increased after atrazine exposure in parkin^−/−^ mice and were not reversed by melatonin supplementation (Fig. 4D). The data indicated that parkin deficiency inhibited mitophagy during atrazine exposure, even after melatonin treatment. More importantly, the activation of parkin-mediated mitophagy is necessary for melatonin to alleviate atrazine-induced renal injury.

### The antisenescence ability of melatonin in parkin^−/−^ mice is absent after exposure to atrazine

**Fig. 5.**
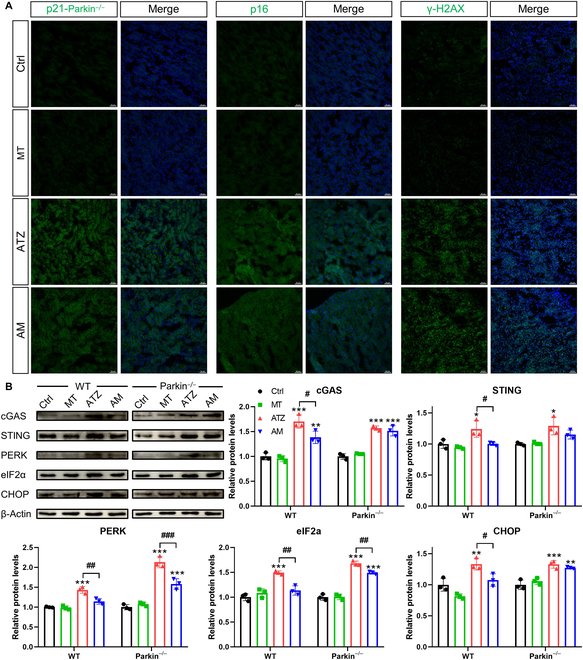
Melatonin alleviates the excessive activation of the cGAS–STING–PERK pathway upon exposure to atrazine through parkin-mediated mitophagy (*n* = 3). (A) Immunofluorescence images of p21, p16, γ-H2AX (green), and DAPI (blue) from parkin^−/−^ mice. Scale bars, 50 μm. (B) Western blotting images and quantitative results of proteins from WT or parkin^−/−^ mice. Statistics data are presented as the means ± SD. Symbol for the significance of differences between the Ctrl group and another group: *P < 0.05, **P < 0.01, and ***P < 0.001. Symbol for the significance of differences between the ATZ group and AM group: *#P* < 0.05, *##P* < 0.01, and *###P* < 0.001.

Since melatonin failed to alleviate renal injury in parkin^−/−^ mice during atrazine exposure, the study further investigates whether melatonin alleviates renal tubular epithelial cell senescence in parkin^−/−^ mice. Atrazine led to an increase in SA-β-gal positively stained areas in parkin^−/−^ mice, compared with wild-type (WT) mice (Fig. 4B). SA-β-gal staining results indicated that melatonin supplementation failed to alleviate atrazine-induced increase in SA-β-gal staining in parkin^−/−^ mice. Western blotting results indicated that p-p53, p21, and p16 levels were increased in kidneys from parkin^−/−^ mice after exposure to atrazine (Fig. 4C). Fluorescent observation also confirmed an increase in fluorescent signals of p21 and p16 (Fig. 5A). Besides, the result displayed higher numbers of γ-H2AX-positive cells in parkin^−/−^ mice following atrazine treatment (Fig. 5A). However, there was no marked alleviation of the above phenomena in parkin^−/−^ mice following melatonin supplementation during atrazine exposure. These findings indicate that the protective role of melatonin in against atrazine-induced renal tubular cell senescence was abolished after parkin deficiency.

### Melatonin inhibits atrazine-induced cGAS–STING–PERK pathway activation and mtDNA release through parkin-mediated mitophagy

As shown in Fig. 5B, Western blotting showed significantly higher levels of cGAS–STING- and endoplasmic reticulum (ER)-associated PERK, eIF2α, and C/EBP homologous protein (CHOP) in atrazine group from WT or parkin^−/−^ mice as compared with the Ctrl group, which indicates that the cGAS–STING–PERK pathway was activated during atrazine exposure. Notably, increased levels of cGAS–STING- and ER-associated proteins triggered by atrazine were significantly attenuated in WT mice but not in parkin^−/−^ mice after melatonin treatment (Fig. 5B). Together with the results, it is suggested that the inhibition of melatonin on the cGAS–STING–PERK pathway was blunted by knockdown on parkin.

**Fig. 6.**
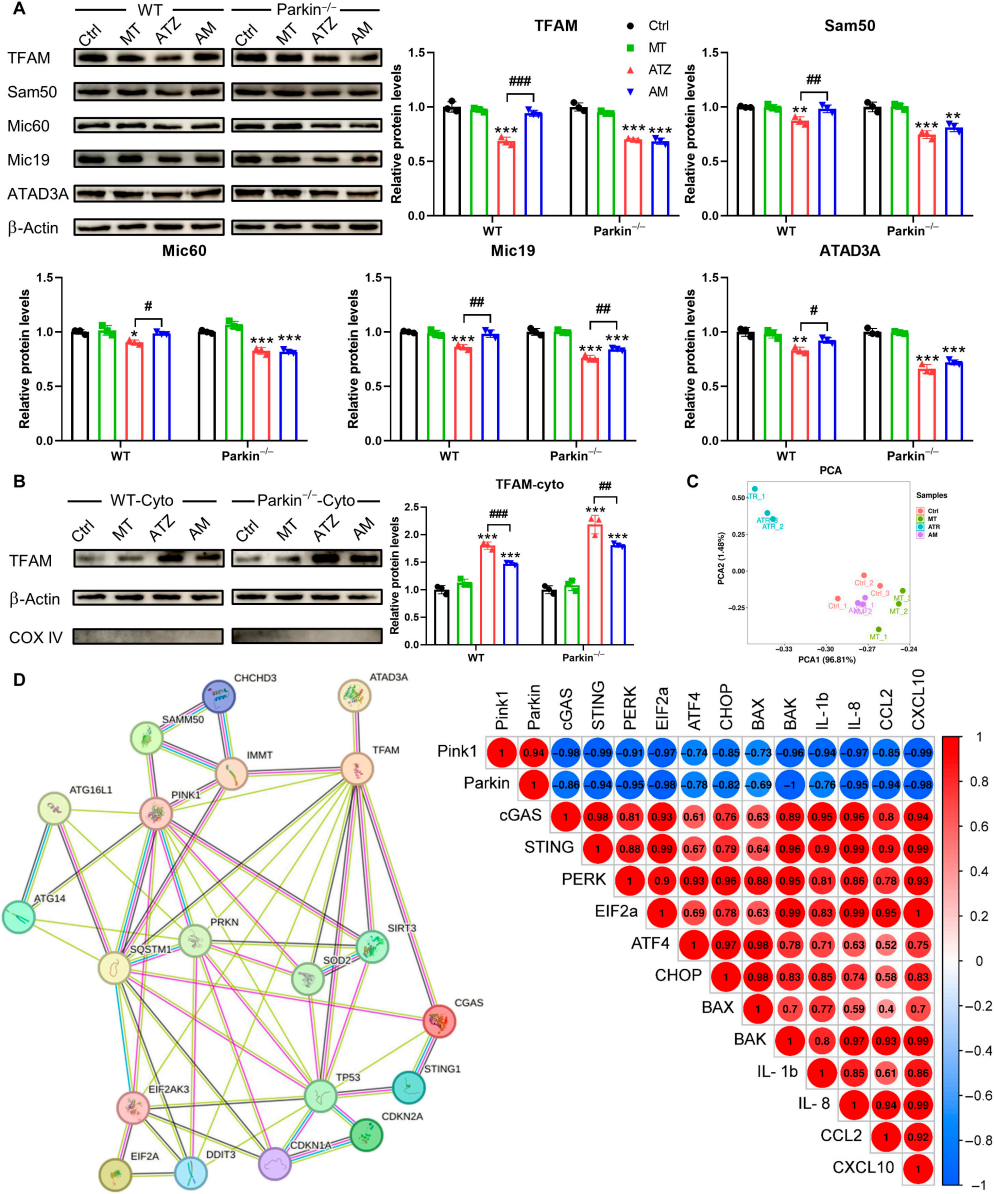
Melatonin maintains the integrity of mitochondrial cristae in a parkin-dependent manner to prevent mtDNA release (*n* = 3). (A and B) Western blotting images and quantitative results of proteins from WT or parkin^−/−^ mice. (C) Perform PCA. (D) Perform PCA and correlational analysis. Statistics data are presented as the means ± SD. Symbol for the significance of differences between the Ctrl group and another group: *P < 0.05, **P < 0.01, and ***P < 0.001. Symbol for the significance of differences between the ATZ group and AM group: *#P* < 0.05, *##P* < 0.01, and *###P* < 0.001.

In WT mice, the decrease in mitochondrial contact site (Mic) 60, Mic19, mitochondrial transcription factor A (TFAM), sorting and assembly machinery 50 (Sam50), and adenosine triphosphatase family AAA domain-containing protein 3 (ATAD3) levels was observed during atrazine treatment, but they were reversed by melatonin (Fig. 6A). The protective roles of melatonin on mitochondrial cristae were constrained by parkin knockdown (Fig. 6A). The cytoplasmic proteins were extracted to assess the distribution of mtDNA. Western blotting results showed that only a few TFAM was observed in cytoplasm of Ctrl and MT groups from WT and parkin^−/−^ mice (Fig. 6B). The level of TFAM in cytoplasm was increased in both WT and parkin^−/−^ mice during atrazine and/or melatonin treatment, the levels of cytoplasmic TFAM in parkin^−/−^ mice during atrazine exposure were higher than those in WT mice (Fig. 6B). Compared with WT mice, parkin^−/−^ hindered melatonin-induced decrease in level of TFAM during atrazine exposure (Fig. 6B). Principal components analysis (PCA) analysis illustrated a marked difference between atrazine group and other groups (Fig. 6C). Correlational analysis revealed a negative correlation between parkin expression and other gene expressions (Fig. 6D and Fig. S3). Taken together, these data indicated that parkin deficiency hinders the decrease in cytoplasmic mtDNA levels by melatonin treatment, thereby leading to a continuous activation of cGAS–STING–PERK pathway.

## Discussion

The use of atrazine is increasing, which poses a threat to public health. Melatonin secreted rhythmically by the mammalian pineal gland and implicated in aging regulation. Preventive measures for potential hazards of atrazine exposure remain inadequate. Damaged mitophagy is considered a trigger for cell senescence. However, the molecular mechanisms underlying senescence and potential interventions are still unclear. The kidneys are very susceptible to senescence because of its high energy demands and frequent exposure to metabolic waste. Aging kidneys predispose people to many kidney diseases, especially chronic kidney disease [30]. Kidney aging not only contributes to functional decline and kidney disease but also leads to a decrease in quality of life. Recent studies have shown that exposure to hazardous substance in the environment is also likely to contribute to organ senescence and diseases [31]. Here, we uncover that blocked parkin-mediated mitophagy is implicated in the progress of renal tubular cell senescence during atrazine-induced renal injury. The melatonin concentration in serum was increased by melatonin treatment in both the MT and AM groups compared with Ctrl group. Furthermore, treatment with melatonin alleviated cell senescence by promoting mitophagy and inhibiting the activation of cGAS–STING–PERK pathway. This study offers novel insights into how to alleviate cellular senescence.

Mitochondria are indispensable for kidneys to meet their energy demands. It is worth noting that damaged electron transport chain in mitochondria is the main sources of ROS. It has been proven that ROS induced DNA damage [32], which further induced senescence by activating cell cycle arrest p53–p21 axis [33]. Oxidative-stress-induced DNA damage is a major factor driving cellular senescence [34]. Sirt3 has been noticed to play a crucial part in prevent oxidative damage by activating SOD2 [35]. The decrease in Sirt3–SOD2 leads to oxidative stress and inflammation [36]. In addition, Sirt3 is also involved in the maintenance of mitochondrial homeostasis and parkin-mediated mitophagy [37]. Numerous studies have suggested that the decrease in Sirt3 has been considered as an indicator of aging [38]. Targeting Sirt3–SOD2 axis is likely to be an effective method for alleviate senescence. Recently, melatonin as a positive modulator of SIRT3 has been considered as a mitochondrial-targeted antioxidant that exerts antioxidant activities in a Sirt3–SOD2-dependent manner [39]. In this research, we observed that melatonin alleviated renal tubular cell senescence by regulating Sirt3–SOD2 axis-mediated antioxidant system.

Accumulating research suggests that mitophagy is the primary manner for removing damaged mitochondria from cells, and blocked mitophagy is associated with many kidney diseases [40]. The protective effect of activating mitophagy on renal injury has been reported by many studies [41]. The role of mitophagy in delaying the process of senescence has also garnered considerable attention. In this study, we found that activated mitophagy is effective in reducing the accumulation of senescent cells during kidney injury. Melatonin treatment restored parkin-mediated mitophagy and autophagy flux during kidney damage, which were indicated by the increase in parkin and decrease in p62 levels. Interestingly, parkin deficiency aggravated renal tubular epithelial cell injury and senescence during exposure to atrazine. Although melatonin partially restored the levels of Sirt3 and SOD2, these changes were not reversed by melatonin treatment. The data indicate that parkin-mediated mitophagy is vital in alleviating senescence by reducing the accumulation of damaged mitochondria. Above all, parkin-mediated mitophagy may be a promising therapeutic target to ameliorate senescence.

Over the past years, the cGAS pathway is traditionally considered to be activated by binding to exogenous double-stranded DNA in cytoplasm and followed by initiating innate immune programs to protect against infection [42]. The mtDNA as a double-stranded DNA can be released from damaged mitochondria into the cytoplasm. TFAM can be used to assess the levels of mtDNA [43]. Recent studies suggest that leaked mtDNA also serves as an important trigger that activates cGAS pathway, which contributes to the inflammatory diseases, neurological diseases, and fibrotic conditions [44]. The aberrantly activated cGAS–STING has been proven to aggravate cell senescence by secreting SASP [45]. ER stress has been linked to many diseases [46–48]. The senescence-promoting effects of ER stress have been proven [49]. Recently, a cGAS–STING–PERK–eIF2α pathway was identified to trigger cell senescence, and the activation of which is independent of unfolded protein response [13]. Despite these findings, it has not been fully elucidated whether mtDNA-induced activation of cGAS is a driving factor in the kidney senescence. Several studies have shown that cellular senescence was observed in cisplatin-induced renal injury [50]. A study found that released mtDNA activated cGAS–STING pathway during cisplatin-induced renal injury [51]. These findings provide an interesting perspective that leaked mtDNA may contribute to senescence. We speculate that atrazine-induced crista damage may result in an enrichment of mtDNA in the cytoplasm, thereby activating cGAS. Consistent with previous study, our result shows that the activation of cGAS–STING–PERK–eIF2α pathway during atrazine exposure was reversed after melatonin treatment, while the reversal was hindered by parkin deficiency. Our study indicates that activated mitophagy plays a central role in inhibiting cGAS–STING pathway during renal tubular epithelial cell senescence.

Within mitochondria, TFAM combines with mtDNA to form a nucleoid structure that stabilizes mtDNA [52]. TFAM also contributes to maintaining mitochondrial function. TFAM is the main regulator responsible for maintaining the transcription and replication of mtDNA, which encodes oxidative-phosphorylation-related proteins for energy generation process [53]. The cells with TFAM deficiency exhibited mitochondria crista disappearance and decreased in adenosine 5′-triphosphate production [54]. The deletion of TFAM increases the levels of mtDNA in cytoplasm [55]. Mice with T cell TFAM deficiency exhibited premature senescence phenotype in various tissues, with heart failure and increased levels of senescence markers. These findings connected TFAM-deficiency-induced mtDNA release with cell senescence. Mic60 and Mic19 are the key components of mitochondrial contact site and cristae organizing system complex, which directly involved in maintaining mitochondrial crista integrity and mtDNA stability along with Sam50 and ATAD3A [56,57]. Recent study also discovered that Sam50–Mic19–Mic60 axis contributes to the formation of crista junction and maintains the integrity of mitochondrial membranes [58]. It has been reported that crista disorganization caused by decrease in Mic60, Mic19, TFAM, Sam50, and ATAD3 levels contributes to mtDNA release [11]. On the basis of our findings that mitochondrial crista structure was damage during atrazine treatment, we supposed that proteins associated with the maintenance of mitochondrial crista structure may play a role in this phenomenon. Our study showed that protein levels of TFAM, Mic60, Mic19, Sam50, and ATAD3A were decreased during exposure to atrazine but reversed by melatonin supplementation. Furthermore, the results demonstrated that the lack of mitophagy prevents melatonin from restoring atrazine-induced declines in levels of key proteins that maintain crista structure.

In conclusion, we demonstrate melatonin supplementation is an effectively therapeutic strategy for alleviating kidney senescence. Blocked mitophagy are required for the progression of cell senescence. Notably, parkin-dependent mitophagy has the potential to become a promising target for mitigating cell senescence.

## Methods

### Animal models

The male WT C57BL/6N mice (6 weeks old) were purchased from Liaoning Changsheng Co. Ltd. Parkin^−/−^ C57BL/6N mice were purchased from Cyagen Biosciences Inc. Atrazine was purchased from Qiaochang Agricultural Group Co. Ltd. Melatonin was purchased from Shanghai Yuanye Bio-Technology Co. Ltd. After 7 d of acclimation, WT and parkin^−/−^ mice were treated with drinking water (Ctrl group), melatonin at a dose of 5 mg/kg per day (MT group), atrazine at a dose of 170 mg/kg per day [atrazine (ATZ) group], and atrazine (170 mg/kg per day) and melatonin (5 mg/kg per day) (AM group) by oral gavage respectively for 28 d (25 mice per group). Atrazine and melatonin were dissolved in drinking water. The dose selections of atrazine and melatonin are based on previous studies [59,60]. All protocols were approved by the Animal Ethics Committee of Northeast Agricultural University.

### LC-MS analysis

LC-MS was used to assay the concentration of melatonin in mouse serum. The male ICR mice serum samples were isolated from the blood that collected after 20 min of gavage treatment. The details were presented in Text S1.

### H&E staining

The kidneys were fixed with 4% paraformaldehyde. Five-micrometer-thick sections were deparaffinized and then stained with H&E. Leica microscope was used for image acquisition. As previously described, kidney pathological changes were scored [61].

### SA-β-gal staining

SA-β-gal staining was performed as prescribed by the manufacture’s protocol (Beyotime, C0602).

### Ultrastructural observation

Preparation of renal samples for transmission electron microscope observation was performed as described [62]. The samples were observed by Hitachi electron microscopy.

### Immunofluorescence

As previously described, frozen sections were fixed by 4% paraformaldehyde for 15 min and then washed with tris-buffered saline for 15 min [63]. The frozen sections were incubated with 10% goat serum that containing 0.3% Triton X-100 for 20 min. After that, sections were incubated with primary antibodies overnight. Subsequently, the sections were washed 3 times with tris-buffered saline, followed by incubation with Alexa Fluor 488 (Beyotime, A0423) for 1 h. The primary antibodies include p21 (ABclonal, AP0083), p16 (ABclonal, AP0083), parkin (Wanleibio, WL02512), LC3 (Wanleibio, WL01506), and γ-H2AX (Beyotime, AF5836). The fluorescence microscope (Leica, Germany) was used to capture images.

### Measurement of oxidative stress biomarker levels

As previously described, the total protein concentration and levels of oxidative stress biomarker were determined using commercial kits (Jiancheng Biotech, China) [64].

### RNA extraction and quantification

The TRIzol was used to extract total RNA. The cDNA was synthesized using commercial kit (TransGen Biotech, China). The primers are shown in Table S4. The relative expression of target gene was normalized to β-actin. Quantitative reverse transcription polymerase chain reaction was performed on QuantStudio (Thermo Fisher Scientific, USA) using commercial kit (TransGen Biotech, China).

### Western blotting analysis

The radioimmunoprecipitation assay lysis buffer and phenylmethylsulfonyl fluoride were used to extract total protein. Tissue mitochondria isolation kit (Beyotime, C3606) was used to extract cytoplasm proteins (without mitochondria). The primary antibodies include β-actin (ABclonal, AC026), p53 (Bioss, bs-8687R), p-p53 (ABclonal, AP0083), p21 (ABclonal, AP0083), p16 (ABclonal, AP0083), Sirt3 (KleanAB, P100413), SOD2 (ABclonal, A19576), pink1 (ABclonal, A11435), Parkin (Wanleibio, WL02512), LC3 (Wanleibio, WL01506), p62 (ABclonal, A7758), Beclin1 (ABclonal, A7353), cGAS (ABclonal, A8335), STING (ABclonal, A21051), PERK (ABclonal, A18196), eIF2α (ABclonal, A21221), CHOP (Wanleibio, WL008800), TFAM (ABclonal, A13552), Sam50 (ABclonal, A3401), Mic60 (ABclonal, A2751), Mic19 (ABclonal, A8584), ATAD3A (ABclonal, A8230), and cytochrome c oxidase (COX) IV (ABclonal, A6564).

### Molecular docking

As previously described, the protein–ligand docking study was performed by AutoDock 4.2 [65]. The PyMOL molecular graphic program was used to illustrate the result.

### Protein–protein interaction analysis

The proteins in the present study were performed a protein–protein interaction analysis using STRING database (Version 12.0).

### Performance of PCA and correlational analysis

PCA and correlational analysis were performed using the OmicStudio tools.

### Statistical analysis

GraphPad Prism 8 was used to perform data analysis. All data were expressed as means ± SD. One-way analysis of variance (ANOVA) followed by Tukey’s test was used to analyze the significance of the differences. *P* < 0.05 is regarded statistically significant.

## Data Availability

The data that support the findings of this study are available from the corresponding author upon reasonable request.
