# Supplementary material for: Melatonin Mitigates Atrazine-Induced Renal Tubular Epithelial Cell Senescence by Promoting Parkin-Mediated Mitophagy
Source: Research (Wash D C). 2024 May 17;7:0378. doi: 10.34133/research.0378 (PMC11098712; doi:10.34133/research.0378)
Supplement: Supplementary 1 — Text S1 Figs. S1 to S3 Tables S1 to S4 [file research.0378.f1.docx]

***Supplemental Materials***

**Melatonin mitigates atrazine-induced** **renal tubular epithelial cell senescence by** **promoting parkin-mediated mitophagy**

Yu-Sheng Shi^1^, Tian-Ning Yang^1^, Yu-Xiang Wang^1^, Xiang-Yu Ma^1^, Shuo Liu^1^, Yi Zhao^1, 2, 3, *^, Jin-Long Li^1, 2, 3, *^

^1^ College of Veterinary Medicine, Northeast Agricultural University, Harbin 150030, P.R. China

^2^ Key Laboratory of the Provincial Education Department of Heilongjiang for Common Animal Disease Prevention and Treatment, Northeast Agricultural University, Harbin 150030, P.R. China

^3^ Heilongjiang Key Laboratory for Laboratory Animals and Comparative Medicine, Northeast Agricultural University, Harbin 150030, P.R. China

***Corresponding author.**

**Yi Zhao**

Address: College of Veterinary Medicine, Northeast Agricultural University, Harbin, 150030, P. R. China

Tel: +86 451 55190407; fax: +86 451 55190407. E-mail address: zhaoyi@neau.edu.cn (Y. Zhao)

**Jin-Long Li**

Address: College of Veterinary Medicine, Northeast Agricultural University, Harbin, 150030, P. R. China

Tel: +86 451 55190407; fax: +86 451 55190407. E-mail address: Jinlongli@neau.edu.cn (J.L. Li)

**Supplementary 1. LC-MS analysis**

In brief, the serum samples were homogenized in 300 μL of methanol and 50 μL of internal standards, swirled and eddied for 1 min, and centrifuged (14,000 g at 4 °C for 10 min) to obtain the supernatant of each sample for LC-MS analysis. The standard solution of melatonin with the concentration levels in the range of 0.05 to 20 ng/mL was prepared, then internal standard was added to the solution, and then homogenization for mass spectrum analysis. The conditions of LC and MS are showed in Table S1-3. Figure S1 shows the chromatograms of a standard solution of melatonin.

**Table S1.** LC conditions.

| **Items** | **Conditions** |
| --- | --- |
| LC system | Waters ACQUITY UPLC I-CLASS |
| Column(s) | Waters ACQUITY UPLC BEH C8 (1.7 μm, 2.1 mm×100 mm) |
| Mobile phase A | 0.5 mM NH_4_F |
| Mobile phase B | 100% Methanol |
| Flow rate | 0.3 mL/min |
| Injection volume | 10.0 µL |
| Column temperature | 50 ℃ |
| Run time | 8 min |
| Gradient | See Table S2 |

**Table S2.** Gradient table for the separation of the melatonin.

| **Time (min)** | **Mobile phase** | |
| --- | --- | --- |
|  | **A（v%）** | **B（v%）** |
| 0 | 60 | 40 |
| 0.5 | 60 | 40 |
| 4 | 45 | 55 |
| 6.5 | 25 | 75 |
| 7.2 | 5 | 95 |
| 8 | 60 | 40 |

**Table S3.** MS Conditions.

| **Items** | **Conditions** |
| --- | --- |
| MS system | Waters XEVO TQ-XS |
| Capillary voltage | 3.0 kV |
| Source temperature | 150 ℃ |
| Cone voltage | 70 V |
| Desolvation temperature | 550 ℃ |

**Figure S1.** Calibration plot and chromatograms.


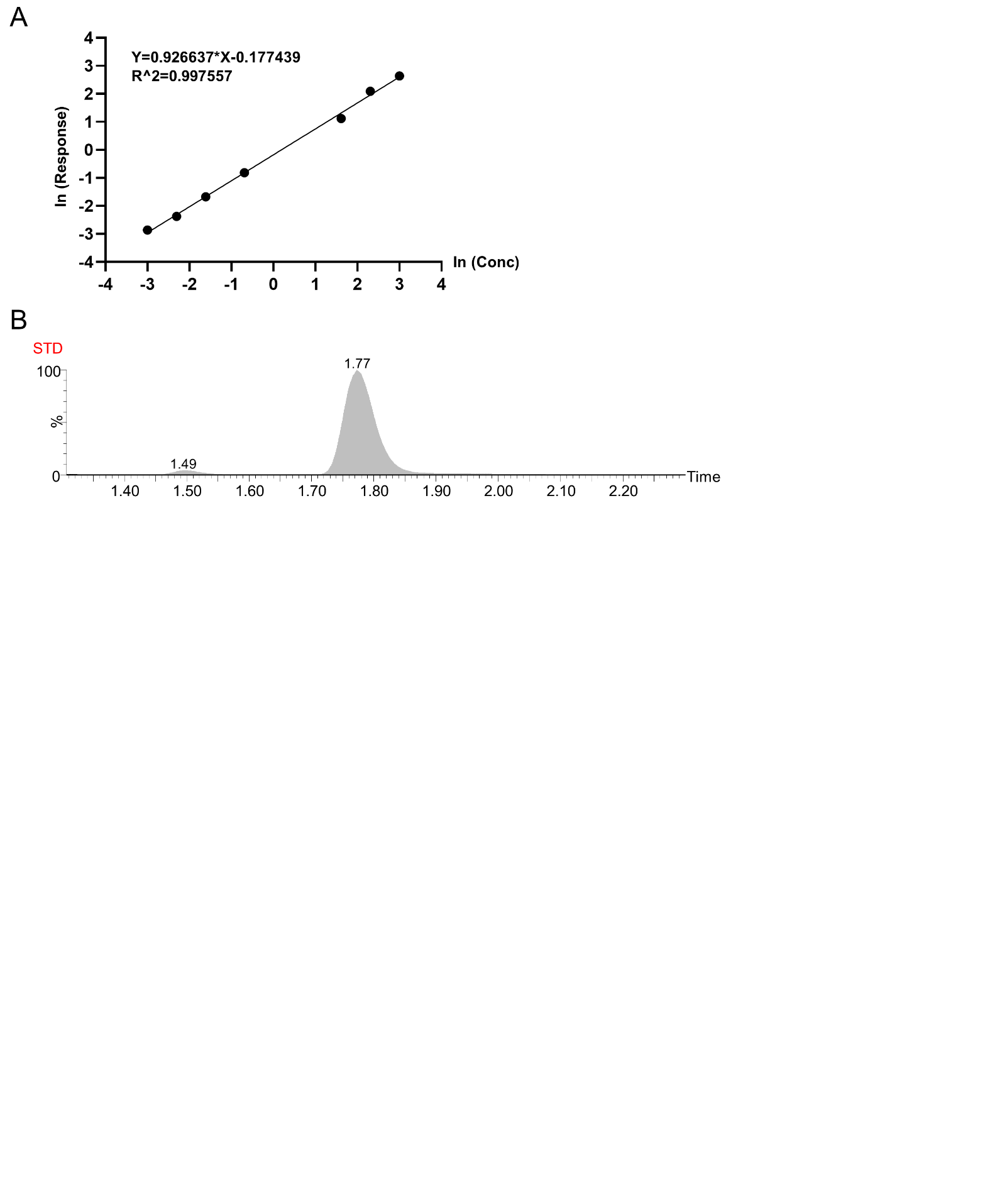


(**A**) Calibration plot for melatonin of concentration levels in the range of 0.05 to 20 ng/mL. (**B**) Chromatograms of a standard solution of melatonin.

**Figure S2.** Characteristics of parkin knockout (parkin^-/-^) mice.


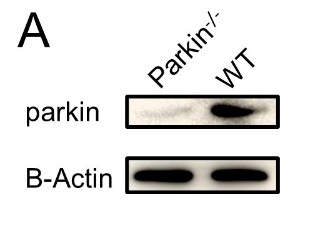


(**A**) Parkin expression in the kidneys from WT and parkin^-/-^ mice.

**Figure S3.** Effects of atrazine and/or melatonin on mRNA expression levels (n=3).


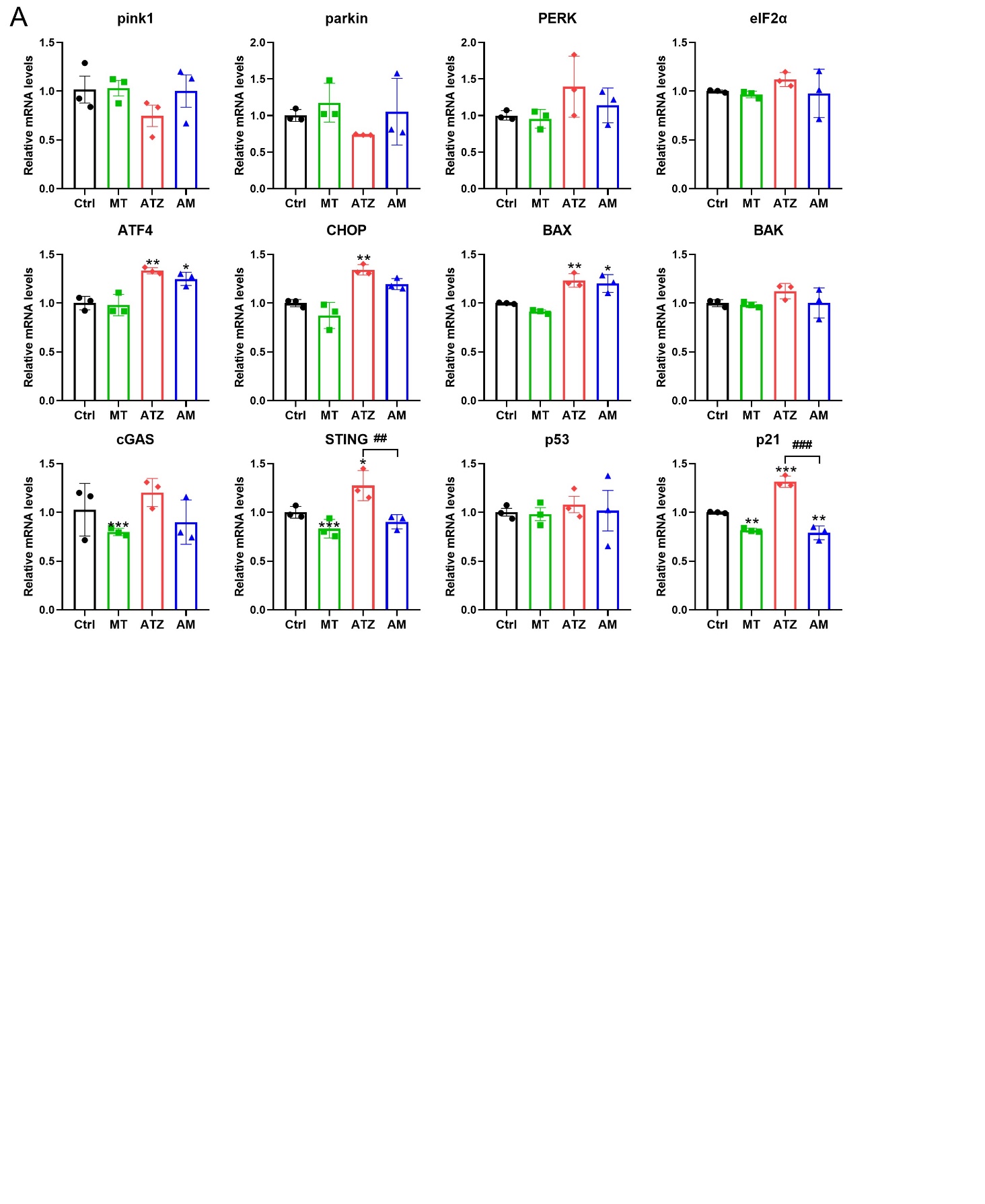


(**A**) Relative mRNA levels in mice kidney. Statistics data are presented as the mean ± SD. Symbol for the significance of differences between the Ctrl group and another group: **P* < 0.05, ***P* < 0.01, ****P* < 0.001. Symbol for the significance of differences between the ATZ group and AM group: *^#^P* < 0.05, *^##^P* < 0.01, *^###^P* < 0.001.

**Table S4.** Sequences of oligonucleotide primers for qRT-PCR.

| **Gene Names** | **Sequence (5' → 3')** | **NCBI reference sequence** | **Amplicon size (bp)** |
| --- | --- | --- | --- |
| β-Actin | AGCCTTCCTTCTTGGGTATGG  GGCATAGAGGTCTTTACGGATGT | NM_007393.5 | 98 |
| Pink1 | CCACCTTTCCCTTTGCCAT  GCTCCTGGCTCATTTTGCTT | NM_026880.2 | 87 |
| Parkin | AAGGAAGTGGTTGCTAAGCGACAG  ATGACTTCTCCTCCGTGGTCTCTG | NM_016694.5 | 156 |
| cGAS | GTTCAAACACAAGAAATGCACTG  GCTGACGGAGTACACAATCCT | NM_173386.5 | 72 |
| STING | TGAAAGGCTCTTCATTGTCTCTT  TGGCATCTTCTGCTTCCTAGA | NM_001289592.1 | 62 |
| PERK | ATCAGCACTTTAGATGGACGAA  AGACCCCACGTCCAAATCCCA | NM_001313918.1 | 81 |
| EIF2α | CAGCTTATAGACCTCCAGCCTT  ATGGCTTGTCACTTCCTGGAT | NM_001005509.2 | 108 |
| ATF4 | ACATTCTTGCAGCCTTTCCC  TCAACTTCACTGCCTAGCTCT | NM_009716.3 | 89 |
| CHOP | CTCGCTCTCCAGATTCCAGT  TGACCACTCTGTTTCCGTTT | NM_007837.4 | 91 |
| BAX | AGACAGGGGCCTTTTTGCTAC  AGACAGGGGCCTTTTTGCTAC | NM_007527 | 137 |
| BAK | ATATTAACCGGCGCTACGACA  TGCCACTCTTAAATAGGCTGGA | NM_007523.3 | 117 |
| IL-1β | ATGCCACCTTTTGACAGTGATG  TGATGTGCTGCTGCGAGATT | NM_008361.4 | 139 |
| IL-8 | GCAACAGAAAGGAAGTGATAGCAG  CCAACAGTAGCCTTCACCCA | NM_011339.2 | 88 |
| CCL2 | GCTACAAGAGGATCACCAGCAG  GTCTGGACCCATTCCTTCTTGG | NM_011333.3 | 106 |
| CXCL10 | AAGTGCTGCCGTCATTTTCT  GTGGCAATGATCTCAACACG | NM_021274.2 | 186 |
| p21 | GCAAAGTGTGCCGTTGTCTC  AAAGTTCCACCGTTCTCGGG | NM_007669.5 | 110 |
| p16 | GAACTCTTTCGGTCGTACCC  ATCTGCACCGTAGTTGAGCA | NM_001040654.1 | 85 |
